# Readmission costs related to intensive care after cardiac surgery. analysis of risk factors and costs within six months after discharge using an administrative registry

**DOI:** 10.1186/2197-425X-3-S1-A65

**Published:** 2015-10-01

**Authors:** A Rossi Zadra, E Caruso

**Affiliations:** AO Città della Salute e della Scienza, Dipartimento di Anestesia e Rianimazione, Torino, Italy; Università di Perugia, Perugia, Italy; CAPP, Modena, Italy

## Introduction

Prolonged stay in intensive care unit (ICU) after cardiac surgery may increase the long-term risk of readmission. Preoperative risk factors, surgical complications, infections and organ failure may need specific intensive support, increasing costs of reimbursement mainly for ICU interventions. Readmission costs over months after discharge may be a dependant of the need of intensive care.

## Objectives

We analyzed the impact of ICU interventions and patients´ conditions on readmission risk and on global costs of reimbursement from the National health service with DRG methodology and assessed the impact of ICU on global costs for complicated cardiac surgery patients.

## Methods

We selected 2067 patients who were admitted to ICUs after cardiac surgery in Regione Piemonte, Italy, in 2009 and analyzed all administrative data listing diagnosis and procedures according to ICD-9CM definitions. Known risk factors for complications and surgical events were selected as ICD-9CM codes. Specific ICU procedures were included if they had impact in DRG calculation. Hospital history was followed for six months after discharge and costs of new admissions were related to selected codes and conditions at the first intervention. We used hazard models and regression analysis to identify ICD9-CM codes that are predictors of readmission and their impact on reimbursement costs, with regard to ICU events.

## Results

528 out of 2067 (25,54%) patients had in total 877 readmissions. In this population hospital length of stay, tracheostomy, heart or kidney failure, infection and the use of IABP or ECMO are strong risk factors for readmission. Tracheostomy accounts for the major increase of costs as DRG consider it an indicator for extensive use of ICU resources. Shock and prolonged mechanical ventilation are inversely related to increased risk of readmission, but they require additional significant expenditure. Some negative findings on the risk of readmission may be explained with increased mortality rate in those patients. Full results are shown in Tables 1-3.

**Table 1:** IMPACT OF ADMISSION CONDITIONS.

	Risk of readmission (logistic regression)	Impact on global costs (linear regression)				
	estimate (logit)	Standard Error	p value	estimate (EUR)	Standard Error	p value
Intercept	-2,416	0,35	< 0.0001	19990	1161	< 0.0001
Female gender	-0,164	0,11	0,14	-79	395	0,84
Age (per single year)	0,009	0,005	0,06	-48	17	0,004
Lenght of stay (days) base reference 0-15 days						
15-28	0,48	0,12	< 0.0001	2170	459	< 0.0001
29-60	0,63	0,20	0,002	5017	789	< 0.0001
>60	1,1	0,41	0.007	16464	1644	< 0.0001
Rehab program	0,46	0,13	0,0005	1036	449	0,021

**Table 2:** IMPACT OF ICU PROCEDURES.

	Risk of readmission (logistic regression)	Impact on global costs (linear regression)				
	estimate (logit)	Standard error	p value	estimate (EUR)	Standard error	p value
Tracheostomy	0,41	0,379	0,28	24367	1472	< 0.0001
Shock (any causes)	-0,44	0,401	0,27	3030	1344	0,02
IABP or ECMO	0,23	0,301	0,44	3318	1117	0,003
Mechanical ventilation >96 hours	-0,48	0,340	0,16	2158	1253	0,08
Dialysis	0,015	0,358	0,97	-433	1374	0,75

**Table 3:** IMPACT OF OTHER CLINICAL CONDITIONS.

	Risk of readmission (logistic regression)	Impact on global costs (linear regression)				
	estimate (logit)	Standard error	p value	estimate (EUR)	Standard error	p value
Infection	0,331	0,322	0,30	-265	1235	0,83
Blood transfusion	1,06	0,321	0,0009	-2847	1292	0,03
Diabetes	0,34	0,164	0,04	-1033	616	0,09
Heart failure	0,34	0,124	0,006	1319	459	0,004
Recent myocardial ischemia	-0,131	0,130	0,32	-1817	460	< 0.0001
Respiratory disease	0,062	0,188	0,74	-634	698	0,36
Kidney disease	0,440	0,202	0,03	-484	788	0,54
Peripheral vascular disease	0,247	0,124	0,05	-1086	454	0,02

## Conclusions

The need of ICU stay and procedures after cardiac surgery may significantly increase the risk of readmission and of reimbursement fee. The ICD-9CM coding system for administrative purposes might be a reliable indicator for the actual clinical risk described in existing literature and predict an increase of expenditure. Health systems should consider ICU costs in allocating resources for cardiac surgery.
